# Odd Integer Quantum Hall States with Interlayer Coherence in Twisted Bilayer Graphene

**DOI:** 10.1021/acs.nanolett.1c00360

**Published:** 2021-05-06

**Authors:** Youngwook Kim, Pilkyung Moon, Kenji Watanabe, Takashi Taniguchi, Jurgen H. Smet

**Affiliations:** † Max-Planck-Institut für Festköperforschung, 70569 Stuttgart, Germany; ‡ Department of Emerging Materials Science, DGIST, 42988 Daegu, Korea; § Arts and Sciences, NYU Shanghai, Shanghai 200122, China and NYU-ECNU Institute of Physics at NYU Shanghai, Shanghai 200062, China; ∥ Research Center for Functional Materials, National Institute for Materials Science, Tsukuba 305-0044, Japan; ⊥ International Center for Materials Nanoarchitectonics, National Institute for Materials Science, Tsukuba 305-0044, Japan

**Keywords:** twisted bilayer graphene, quantum Hall effect, Bose−Einstein condensation, interlayer coherence

## Abstract

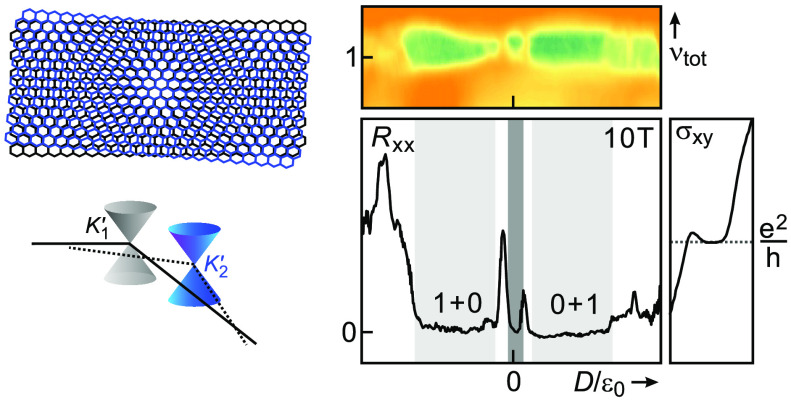

We report on the quantum Hall effect in two stacked graphene layers rotated by 2°. The tunneling strength among the layers can be varied from very weak to strong via the mechanism of magnetic breakdown when tuning the density. Odd-integer quantum Hall physics is not anticipated in the regime of suppressed tunneling for balanced layer densities, yet it is observed. We interpret this as a signature of Coulomb interaction induced interlayer coherence and Bose–Einstein condensation of excitons that form at half filling of each layer. A density imbalance gives rise to reentrant behavior due to a phase transition from the interlayer coherent state to incompressible behavior caused by simultaneous condensation of both layers in different quantum Hall states. With increasing overall density, magnetic breakdown gains the upper hand. As a consequence of the enhanced interlayer tunneling, the interlayer coherent state and the phase transition vanish.

The study of two-dimensional electron systems in close proximity has been a very rewarding subject in the III–V semiconductor community. The observation of Bose–Einstein condensation is an example of one of the most prominent outcomes.^1^ The rich physics in these coupled electron systems stems from the additional degrees of freedom they offer. Tunneling allows for the transition of charge carriers between the layers, and the interlayer Coulomb interactions compete with intralayer interactions. Both can be tuned by changing the characteristics of the tunnel barrier and the interlayer separation. For small layer spacing, interlayer tunneling mixes the levels of each layer, forming symmetric and antisymmetric states. In III–V semiconductors, the resulting energy gap is usually smaller than the cyclotron energy at the typical magnetic fields, but comparable with the interlayer Coulomb interaction energy. This often leads to incompressible ground states distinct from those found in single layer systems. It causes a magnetic field driven collapse of the tunneling induced energy gap as well as the formation of a Bose–Einstein condensate for instance at half filling of each layer, heralded by quantum Hall behavior at odd integer filling.^2−7^

The state-of-the-art mobilities offered by GaAs double layer systems were instrumental for the discovery of this condensate as well as Josephson-like tunneling behavior and vanishing Hall resistance in counter-flow experiments, considered a signature of excitonic superfluidity.^3,4^ More recently, the flexibility in device design and sample quality offered by graphene layers separated by an insulator with atomic flatness and thickness control through van der Waals stacking and encapsulation has caused the community to revisit this field in order to benefit from the exceptional charge carrier tunability offered and the expected enrichment of the physics due to the additional valley degree of freedom. Analogous phenomena such as those observed in GaAs were first reported, including the quantum Hall state at total filling 1 due to Bose–Einstein condensation, as well as vanishing Hall resistance in counter-flow experiments.^8,9^ However, more exotic interlayer paired states, like the ν_tot_ = 1/3 state, have also been discovered.^10,11^

Here, we address the extreme regime of sub-nanometer layer spacing and yet strongly suppressed interlayer coupling or tunneling, which has not been at the focal point so far. To this end, we exploit the virtues of twisted graphene layers without an insulating layer in between. As a result of the twist, the Dirac cones from each layer are displaced in momentum space as illustrated in Figure 1a, which can strongly suppress the interlayer coupling or tunneling.^12^ Near the so-called magic angle of 1.05°, the band dispersion becomes flat and both layers are always strongly coupled. This gives rise to a rich phase diagram with ferromagnetic, correlated insulating and superconducting ground states.^13−19^ Of interest here are larger twist angles that produce an energy dispersion in the reduced Brillouin zone closely resembling that of graphene with a van Hove singularity due to the merger of the displaced Dirac cones of the original layers (see Figure 1a). The singularity occurs at an energy no longer set by the graphene hopping parameter (about 3 eV), but a much lower twist angle controlled energy. Prior to reaching the van Hove singularity, magnetic breakdown due to the uncertainty in reciprocal space in a magnetic field^20−22^ allows tunneling across momentum gaps between the disjoint and closed Fermi surfaces that form in the reduced Brillouin zone (Figure 1b). The original Dirac cone states at the displaced *K*-symmetry points give rise to a band with closed Fermi surfaces encircling the *K̅* and *K̅*′ points of the reduced Brillouin zone (solid circles). The coloring refers to the layer the states originate from (black for layer 1 and blue for layer 2). The same holds for the Dirac cone states at the displaced *K*′-symmetry points, however with an interchanged location in the reduced zone (dotted Fermi surfaces). The momentum gaps at the reduced zone boundary shrink with increasing density (Figure 1b), and the tunneling probability rises. It is this mechanism that accounts for enhanced “hybridization” or “coupling” between the different layer states with increasing density. The system can then no longer be regarded as composed of two separate layers. When the chemical potential crosses the van Hove singularity, the Fermi surfaces of each band merge into a single Fermi surface, effectively removing the layer degree of freedom all together.

**Figure 1:**
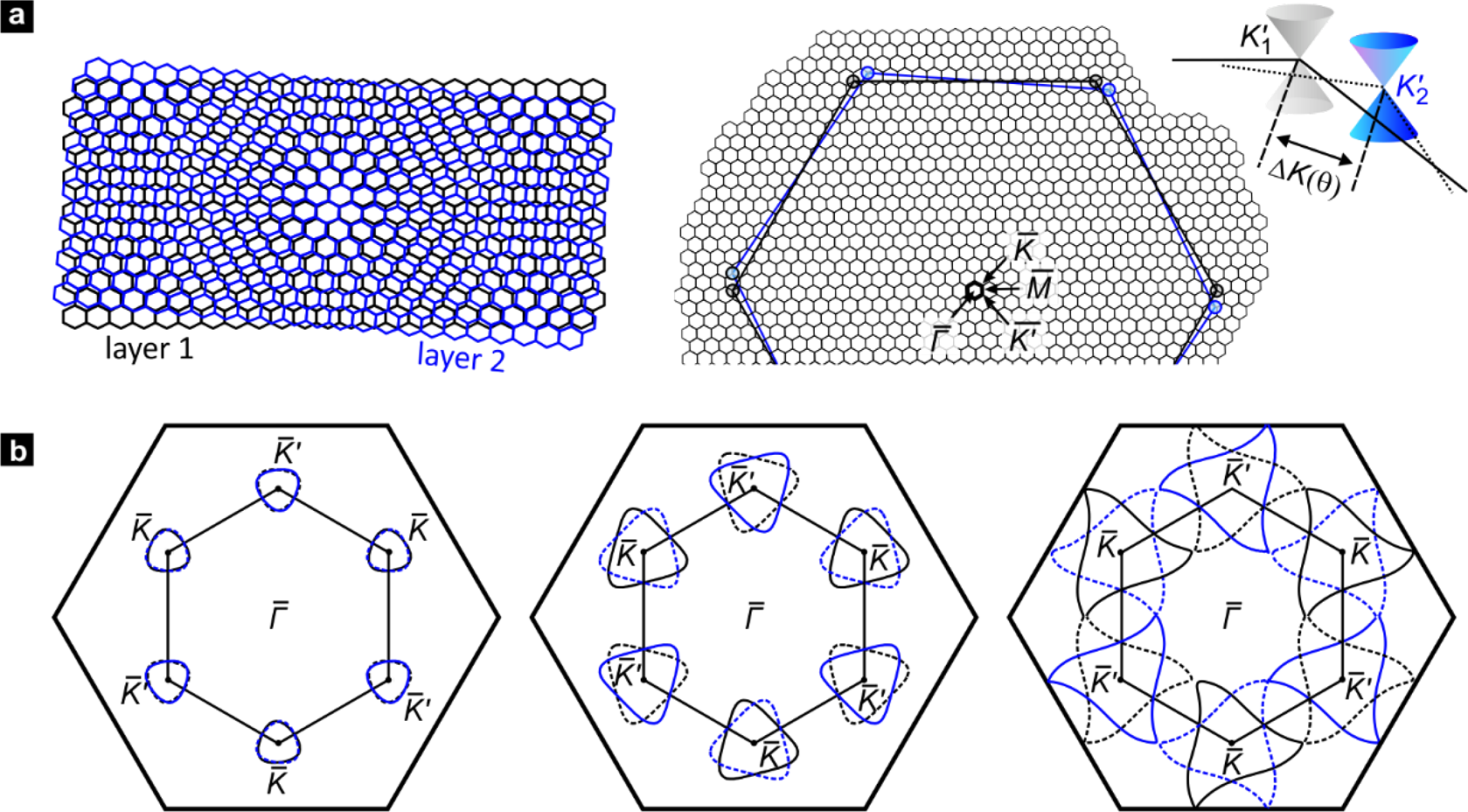
Band structure of twisted bilayer graphene. (a) Real-space lattice structure of two graphene layers twisted by an angle θ (left, black – reference layer, blue – twisted layer). The twist in real space causes a displacement of the Dirac cones of the two layers in reciprocal space (right) by an amount determined by the twist angle θ. The black and blue large hexagons indicate the first Brillouin zone of the two layers. The energy where both Dirac cones would intersect increases with increasing twist angle. Also shown is a schematic of the reduced Brillouin zone and the notation used for the symmetry points. (b) For a twisted bilayer, there are two bands. Each of them generates closed isoenergetic contours around the *K̅* and *K̅*′ symmetry points below the van Hove singularity. The three panels correspond to three different energies. The Fermi contours associated with the first band are plotted with solid lines. Fermi contours that stem from band 2 are plotted with dotted lines. The coloring of the Fermi surfaces indicates whether the states originate from the Dirac cone of the first (black) or second layer (blue).

At small angles above the magic angle, the chemical potential can easily be lifted up to this energy with conventional gating techniques.^23,24^ Hence, it is possible to explore the regime of essentially decoupled layers at low densities, weakly tunnel coupled layers at intermediate densities and strongly coupled layers when the chemical potential comes closer to the van Hove singularity. All this can be accomplished at the turn of a gate voltage knob. Devices with twist angles between 2° and 3° represent the sweet spot, because the Fermi velocity in the low energy regime is significantly reduced compared to devices with larger twist angles. The enhanced density of states boosts the importance of Coulomb interactions. Moreover, for twist angles exceeding 5° the overlap matrix element^25^ or interlayer transitions between eigenstates of the opposite valleys *K* and *K*′, i.e., between the two Fermi surfaces encircling either the *K̅* and *K̅*′ points of the reduced Brillouin zone (Figure 1b), grows rapidly, disrupting the separate layer picture.

As opposed to previous studies focusing either on large twist angles^26−28^ or the magic angle,^13−19^ here we address the transport properties of a device with a twist angle of 2° to search for Bose–Einstein condensation related quantum Hall states for the previously mentioned reasons. For this angle, the van Hove singularity energy is only about 25 meV.^23,24^ A device schematic and image are shown in panels a and b of Figure 2. A bottom and top gate enable the application of a displacement electric field *D*. Further details of the device and gating characteristics are deferred to the Supporting Information. A color map of the longitudinal resistance recorded across the plane spanned by the total density *n*_tot_ and *D*/ε_0_ is plotted in Figure 2c. Here, ε_0_ is the permittivity of vacuum. Key features are highlighted: the charge neutrality point at *n*_tot_ = 0, and the two satellite peaks signaling full occupation or depletion of the Moiré potential induced miniband centered around zero energy. The latter appear at a hole and electron density of approximately 8.5 × 10^12^ cm^–2^ (section S2, Supporting Information). The density accommodated by the miniband yields the area of the reduced Brillouin zone from which it is possible to determine the twist angle of 2°. The same twist angle can also be obtained from the Landau fan chart.

**Figure 2:**
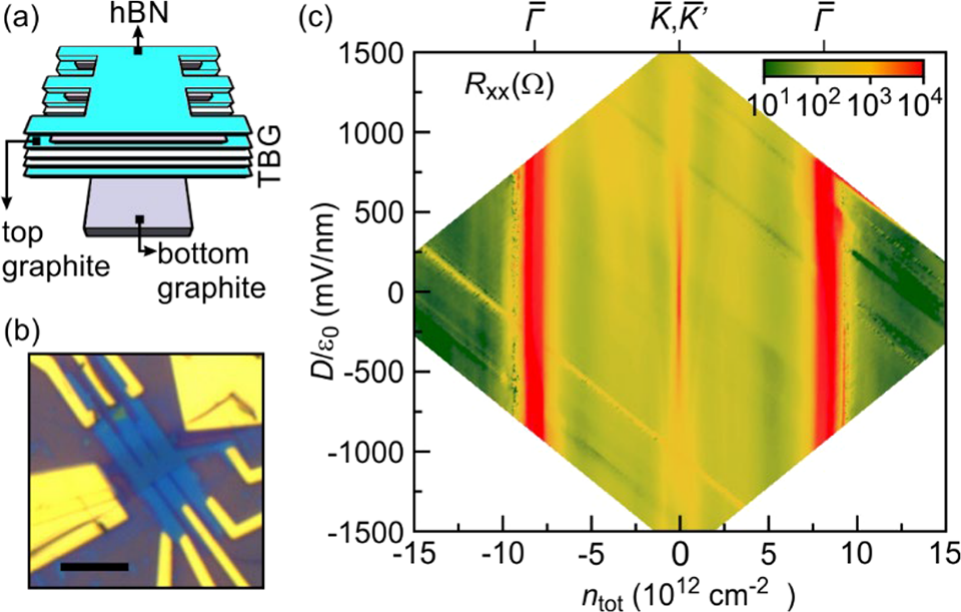
(a) Schematic of the twisted bilayer graphene device consisting of a graphitic bottom gate, a hBN dielectric layer, the twisted bilayer, a hBN dielectric layer, a graphitic top gate, and finally a hBN cap layer. (b) Optical microscope image of the device. The scale bar corresponds to 5 μm. (c) Color map of the longitudinal resistance, *R*_*xx*_, in the plane spanned by the carrier density and the displacement field for zero magnetic field. The narrow red region near zero density corresponds to the conventional resistance peak at charge neutrality when the chemical potential crosses the Dirac point at the *K̅* and *K̅*′-symmetry point. Secondary charge neutrality peaks also appear as red regio*n*s near *n*_tot_ = ±8 × 10^12^ cm^–2^ and signal that the miniband formed due to Moiré superlattice potential induced zone folding is either completely emptied or filled at the *Γ̅*-point.

In Figure. 2c, the charge neutrality peak at the Dirac point rapidly diminishes in amplitude (from red to yellow) when applying an electric field across the layers, while the strength of the two satellite peaks remains nearly constant. This behavior reflects that when the chemical potential is near zero energy, the two layers are decoupled due to the momentum mismatch of the displaced *K* and *K*′ symmetry points for the two layers. The displacement field does not cause a band gap as in Bernal stacked bilayer graphene, but shifts the chemical potential with respect to the Dirac zero energy points of the two graphene sheets in opposite direction. There is then a nonzero density of states in both graphene layers at the chemical potential. Holes accumulate in one layer and electrons in the other layer resulting in low resistivity, while the net density remains zero. In contrast, near the secondary charge neutrality points the resistivity is insensitive to the displacement field. This comes as no surprise, since beyond the van Hove singularity, the layers are strongly coupled and the resistance peak results from the twist angle induced bandgap separating adjacent minibands. This behavior due to miniband formation is consistently observed both in magic angle devices as well as in a 2° twisted device.^12−19,23−25,29^

In Figure 3a, the longitudinal magnetoresistance and the Hall conductance are plotted when the sample is exposed to a fixed perpendicular magnetic field of 3T. At zero displacement field and when the two layers are decoupled, it is anticipated that the sequence of observable QH states is governed by the 8-fold degeneracy due to the spin, valley, and layer degrees of freedom at total filling ν_tot_ = ..., −20, −12, −4, +4, +12, +20,···. This is confirmed in the experimental trace of Figure 3a. A color rendition of the longitudinal resistance across the plane spanned by the displacement electric field and either the total density or total filling is shown in panel b of Figure 3. At larger densities, both layers hybridize due to magnetic breakdown as the momentum gaps between the Fermi surface orbits shrink with increasing carrier density. The layer degree of freedom is effectively removed when the chemical potential approaches the van Hove singularity due to the Lifshitz transition from two to one Fermi surface per band. The 8-fold degeneracy is replaced by a 4-fold degeneracy. Experimental manifestations of this can be seen in Figure 3a. The longitudinal resistivity develops in the absence of a displacement field additional minima at ν_tot_ = ±24 and ±32, marked by blue arrows in Figure 3a. This coincides with anomalous behavior of the Hall conductance due to coexistence of electrons and holes when the Lifshitz transition occurs.^24^ This transition is stretched on the *n*_tot_ abscissa, since the density of states is large at the singularity. When applying a nonzero displacement, these minima become more pronounced in either direction, and no transition occurs. Hence, the displacement field merely improves the resolution of the layer degeneracy removal, and the two layers act as one layer irrespective of the size of the displacement field in the range accessible in our experiment. This can be seen in Figure 3b along the lines of constant ν_tot_ = ±24 and ±32. This behavior is distinct from that at other, lower filling factors, i.e., at significantly lower total densities, where a multitude of displacement field induced transitions can be observed. We assert that this difference stems from the decoupled or weakly coupled nature of the layers at these lower fillings/densities, as will be discussed in detail below. The Hall conductance also develops additional features at fillings ν_tot_ = ±24 and ±32; however, the observation of a clear transition from 8e^2^/h steps to 4e^2^/h steps is hampered by the overall sign reversal of the Hall conductance due to the conversion of the charge carriers from holes to electrons or vice versa, as the chemical potential crosses the van Hove singularity energy.

**Figure 3:**
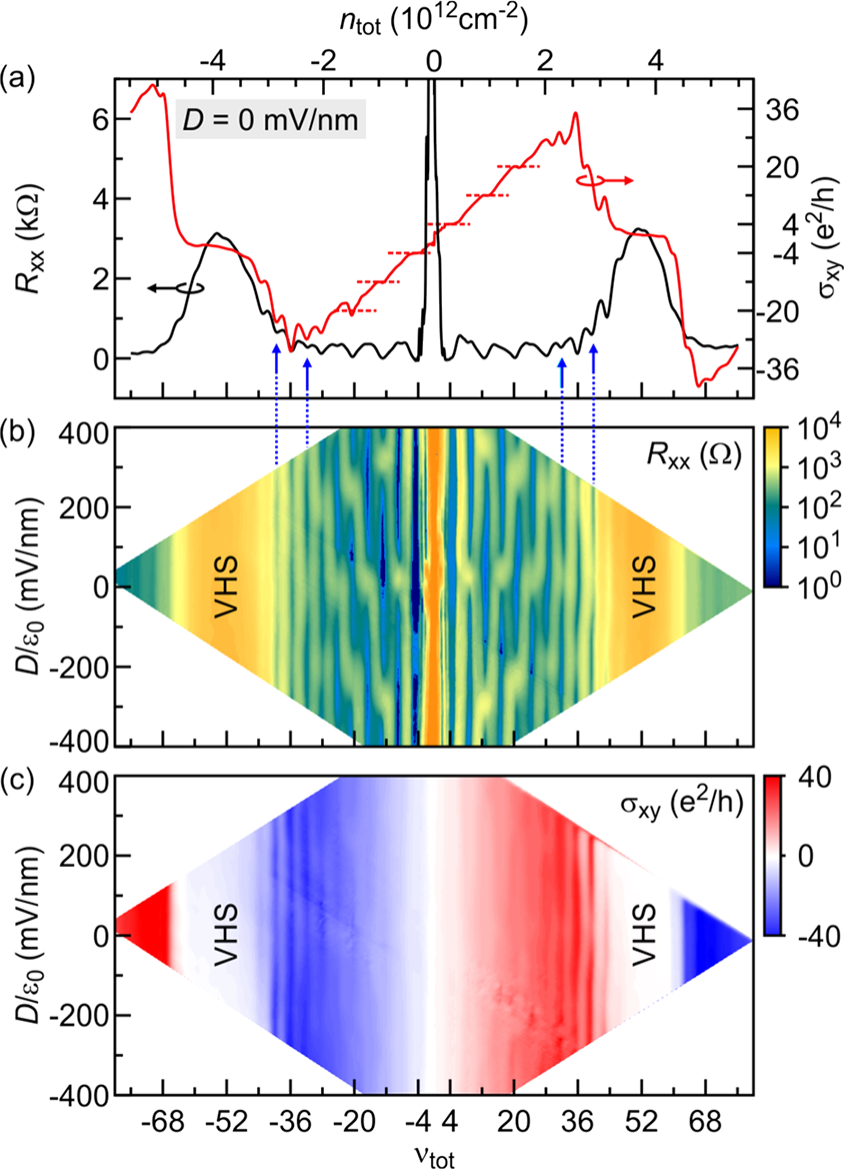
(a) Longitudinal resistance, *R*_*xx*_, and Hall conductance, σ_*xy*_, as a function of the total filling factor or density at a fixed magnetic field of 3 T and in the absence of a displacement field. Red dashed lines highlight the 8e^2^/h steps in the Hall conductance, for instance, at ν_tot_ = ±4, ±12, and ±20. Blue arrows and dotted lines point to incipient quantum Hall behavior when the chemical potential approaches the van Hove singularity and the states of both layers hybridize so the system effectively behaves as a single layer. (b) Color map of *R*_*xx*_ in the (ν_tot_, *D*/ε_0_)-plane. The magnetic field is fixed at 3 T. (c) Same, but for σ_*xy*_.

Figure 4 shows a color map of the longitudinal resistance as a function of total filling factor and displacement field as well as single line traces of both the longitudinal resistance and Hall conductance for the low density/low filling regime when the layers are decoupled or weakly coupled only. In the absence of a displacement field, i.e., equal densities in both layers, quantum Hall behavior is only anticipated at even total filling when both layers simultaneously condense in the same quantum Hall state, for instance, ν_tot_ = ±2 for equal filling one of both layers. At nonzero displacement field and fixed total filling, charges are redistributed among the layers. Each layer may then condense in a different quantum Hall state, which would also produce overall incompressible behavior with vanishing resistance. Examples of this can be seen in Figure 4a and c. At ν_tot_ = 2, the experiment in panel a matches expectations. For *D* = 0, the QHE develops, since both layers take on filling 1. As the displacement field is changed, the top or bottom layer accumulates more charges until it reaches filling 2, while the other layer is emptied resulting in reentrant behavior. This in principle can continue further with hole fillings for one layer at even larger *D* resulting in additional transitions. The schematic on the right in panel a of Figure 4 highlights the integer filling of the top and bottom layer at fixed total integer filling for the observed QH-minima in the experimental data on the left. A similar sequence of transitions between QH states also occurs at ν_tot_ = 1 (Figure 4a) and can be understood in this picture of decoupled layers. However, contrary to expectation, also for *D* = 0 at ν_tot_ = 1, the bilayer system turns incompressible (green dot in the schematic in panel a) as seen in the single line traces of *R*_*xx*_ and σ_*xy*_ recorded at 10 T in Figure 4b as well as at fixed ν_tot_ = 1 for different *B*-fields in Figure 4c (highlighted by the star). In large twist angle bilayer graphene, only even integer QH states appear for *D* = 0. Odd integer QH physics only emerges for *D* ≠ 0.^26^ This unexpected ν_tot_ = 1 state at *D* = 0 in our sample is well separated from the QH behavior due to condensation of both layers into their own quantum Hall state by resistance peaks that appear on either side when moving away from *D* = 0. The resistance peaks are followed by reentrant quantum Hall behavior, because the top and bottom layer approach fillings ν_top_ = 1 and ν_bot_ = 0 or vice versa. All QH states including the *D* = 0 state, and the transitions become more pronounced with increasing *B*-field (Figure 4c). We have focused here on electron occupation only. Hole transport is discussed in the Supporting Information, but it is of lesser quality.

**Figure 4:**
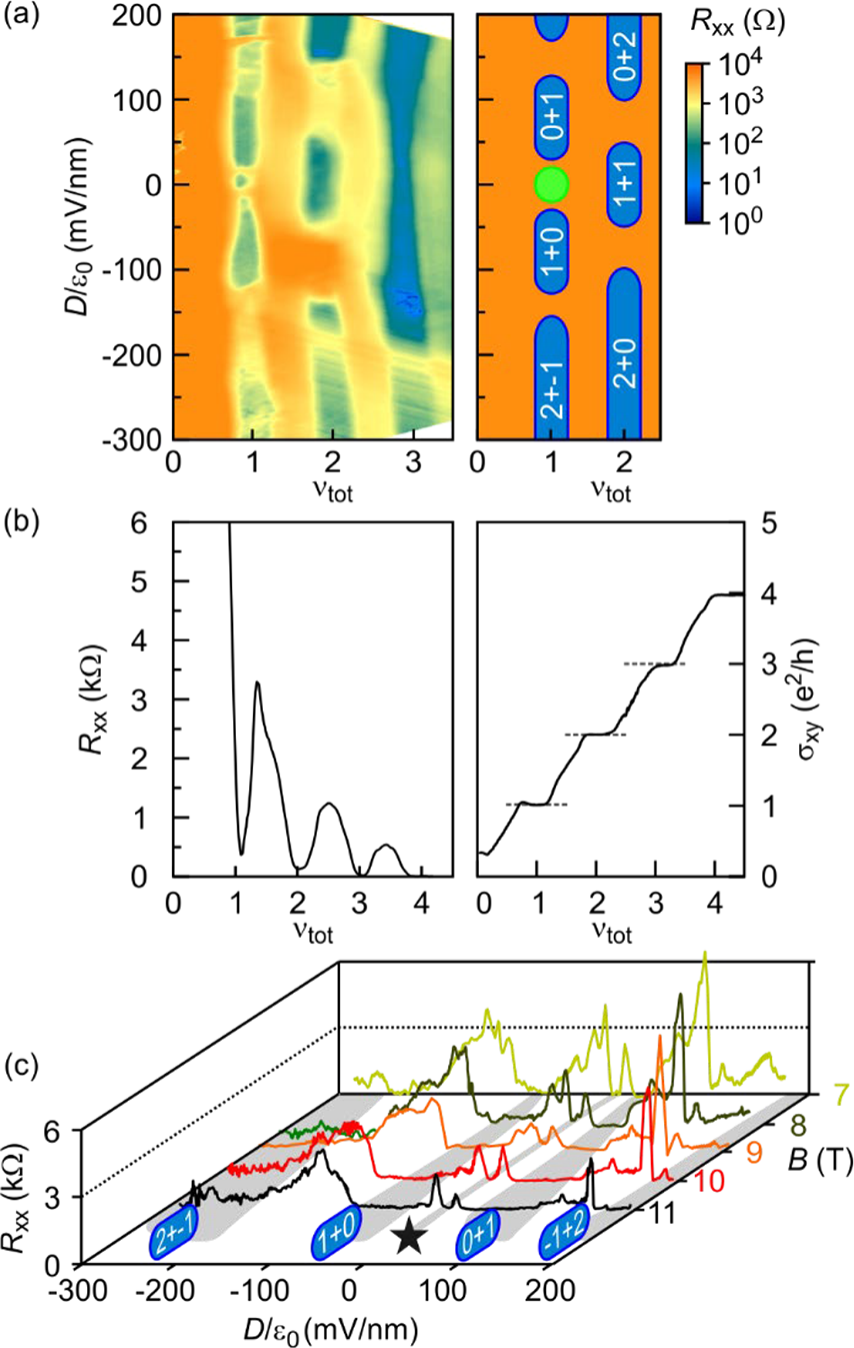
(a) Color map of *R*_*xx*_ (left) as a function of the displacement field and the total filling factor for *B* = 10 T. The schematic on the right indicates regions where quantum Hall behavior is observed due the simultaneous formation of an integer quantum Hall state in each of the layers. The integer numbers correspond to the filling factor of the top (ν_top_) and bottom (ν_bot_) layers. (b) (Left) Longitudinal resistance, *R*_*xx*_, and (Right) Hall conductivity, σ_*xy*_, as a function of ν_tot_ at 10 T when *D*/ε_0_ = 0 mV/nm. Gray dashed lines mark symmetry broken states. (c) Traces of the longitudinal resistance as a function of *D*/ε_0_ for different fixed magnetic fields (different colors). All data were recorded at *T* = 1.3 K.

In principle, a ν_tot_ = 1 QH state with *D*/ε_0_ close to 0 mV/nm may arise either because of interlayer Coulomb interaction driven Bose–Einstein condensation or because a genuine fractional quantum Hall state forms in each of the layers separately, such as, for instance, at ν_top_ = 1/3 and ν_bot_ = 2/3 or ν_top_ = ν_bot_ = 1/2. This fractional quantum Hall scenario to produce a ν_tot_ = 1 for *D* = 0 can, however, be discarded. Our device shows no hint of fractional QH behavior even at 14 T and 30 mK. A half-filled fractional quantum Hall state in an isolated graphene monolayer has only been observed so far when the valley degeneracy is lifted as a result of symmetry breaking by a Moiré superlattice potential imposed by an adjacent hBN film or at magnetic fields exceeding 30 T.^30^ Neither condition, however, applies here. Hence, we conclude that the interlayer Coulomb interaction plays a crucial role in reestablishing interlayer coherence in the twisted bilayer. Without interactions, it is absent in essence because of the twist induced moment mismatch of the Dirac cones of the two layers. This is manifested in the absence of odd integer quantum Hall states and odd numerator fractional quantum Hall states in bilayers with large twist angle when *D*/ε_0_ = 0 mV/nm.^27^ Our experimental data demonstrate that interlayer coherence and the accompanied exciton condensation can be restored at balanced densities at lower twist angle, resulting in ν_tot_ = 1 quantum Hall behavior. Note that with increasing density or field, i.e., as the chemical potential approaches the van Hove singularity, the states of both layers start to hybridize and the system’s behavior should return to that of a single layer. With increasing total density and fields above 12 T, the ν_tot_ = 1 QH state no longer shows clear reentrant behavior when moving away from *D* = 0. The flanking resistance peaks diminish in strength (see Figure S2 of the Supporting Information). In this regime, interlayer coherence no longer needs to be invoked to account for the observed physics.

The quantum Hall effect also appears at ν_tot_ = 3 for balanced layer densities. The emergence of this ground state and its evolution at lower fields are illustrated in Figure 5. The longitudinal resistivity around ν_tot_ = 3 filling as a function of *D*/ε_0_ is plotted as a color map in panel a for different magnetic field values (3–6 T). At low fields, this ν_tot_ = 3 QH state centered around *D* = 0 undergoes again a transition heralded by longitudinal resistance peaks with ascending magnitude of the displacement field followed by reentrant quantum Hall behavior because the two layers condense simultaneously in two different quantum Hall states (here filling 1 or 2 and vice versa for the other layer). At higher field, such transition peaks vanish, completely analogous to the behavior at ν_tot_ = 1. This can be seen in the data recorded at 12 T in Figure S2. The fields at which the layer coherent state associated with exciton condensation disappears is much lower (about three times) for ν_tot_ = 3 than for ν_tot_ = 1. This, however, should not come as a surprise, as this corresponds to about the same total density where single layer behavior is recovered.

**Figure 5:**
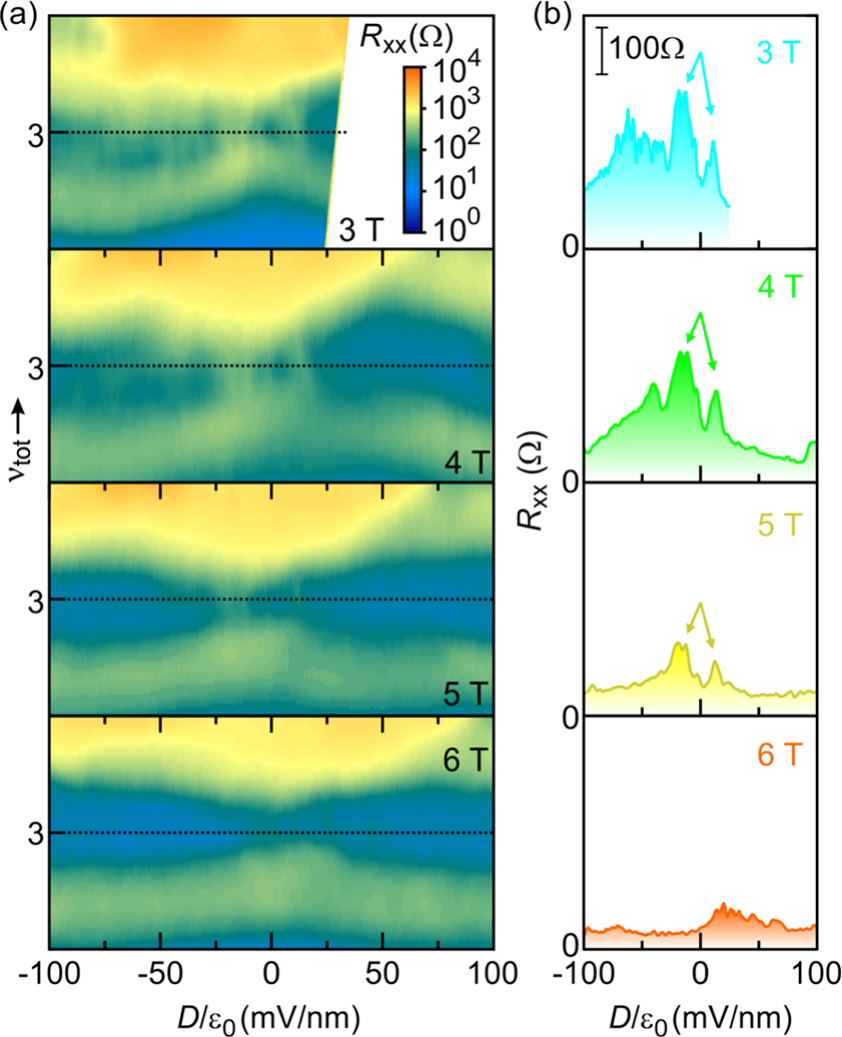
(a) Color map of *R*_*xx*_ near the ν_tot_ = 3 state at magnetic fields from 3 to 6 T (1 T steps). Black dotted lines indicate ν_tot_ = 3. The range of total filling factor covered is 2.5 to 3.5. (b) Line-cuts at fixed ν_tot_ = 3 along the black dotted lines in (a). All windows in (b) have the same *y*-axis. The vertical bar corresponds to 100 Ω. Arrows mark the resistance peaks signaling the transition from the interlayer coherent ground state to a ground state involving incompressible behavior in each layer separately. These transitions are distinguishable up to about 5 T.

In summary, the peculiar dispersion of twisted bilayer graphene consisting of two displaced Dirac cones, that are each composed of states belonging to one of the constituent layers and that hybridize at higher energy due to magnetic breakdown, offers the unique opportunity to modify the effective interlayer coupling strength or tunneling from very weak to strong simply via the mechanism of magnetic breakdown when tuning the density. The layer degree of freedom is effectively removed as the chemical potential approaches the van Hove singularity. These properties have been exploited here to investigate the appearance and evolution of odd integer quantum Hall states with layer tunneling strength. In the regime of low density when the chemical potential is far away from the van Hove singularity and the layers are weakly coupled, the odd integer quantum Hall effect is observed and attributed to interlayer coherence and the formation of a Bose–Einstein condensate of excitons formed by holes and electrons in half filled Landau levels of the two layers. As the chemical potential is raised and the states of both layers hybridize, conventional single layer quantum Hall physics is restored instead.

## Supporting Information Available

Sample fabrication and gating details, Top- and back-gate voltage dependence of the longitudinal resistance, Weakening of the reentrant quantum Hall behavior at ν_tot_ = 1, Effect of the graphene electrode with asymmetric gate tuning, Magnetic breakdown in a bilayer with a 2° twist angle (PDF)

## References

[ref1] Eisenstein J. P.; Boebinger G. S.; Pfeiffer L. N.; West K. W.; He S. New fractional quantum Hall state in double-layer two-dimensional electron systems. Phys. Rev. Lett. 1992, 68, 1383–1386. 10.1103/PhysRevLett.68.1383. 10046152

[ref2] Wiersma R. D.; Lok J. G. S.; Kraus S.; Dietsche W.; von Klitzing K.; Schuh D.; Bichler M.; Tranitz H. P.; Wegscheider W. Activated transport in the separate layers that form the ν_T_=1 exciton condensate. Phys. Rev. Lett. 2004, 93, 266805 10.1103/PhysRevLett.93.266805. 15698006

[ref3] Kellogg M.; Eisenstein J. P.; Pfeiffer L. N.; West K. W. Vanishing Hall resistance at high magnetic field in a double-layer two-dimensional electron system. Phys. Rev. Lett. 2004, 93, 036801 10.1103/PhysRevLett.93.036801. 15323851

[ref4] Tutuc E.; Shayegan M.; Huse D. Counterflow measurements in strongly correlated GaAs hole bilayers: Evidence for electron-hole pairing. Phys. Rev. Lett. 2004, 93, 036802 10.1103/PhysRevLett.93.036802. 15323852

[ref5] Eisenstein J. P.; Macdonald A. H. Bose–Einstein condensation of excitons in bilayer electron systems. Nature 2004, 432, 691–694. 10.1038/nature03081. 15592403

[ref6] Zhang D.; Huang X.; Dietsche W.; von Klitzing K.; Smet J. H. Signatures for Wigner crystal formation in the chemical potential of a two-dimensional electron system. Phys. Rev. Lett. 2014, 113, 076804 10.1103/PhysRevLett.113.076804. 25170727

[ref7] Zhang D.; Dietsche W.; von Klitzing K. Anomalous interlayer transport of quantum Hall bilayers in the strongly Josephson-coupled regime. Phys. Rev. Lett. 2016, 116, 186801 10.1103/PhysRevLett.116.186801. 27203339

[ref8] Liu X.; Watanabe K.; Taniguchi T.; Halperin B. I.; Kim P. Quantum Hall drag of exciton condensate in graphene. Nat. Phys. 2017, 13, 746–750. 10.1038/nphys4116.

[ref9] Li J. I. A.; Taniguchi T.; Watanabe K.; Dean C. R. Excitonic superfluid phase in double bilayer graphene. Nat. Phys. 2017, 13, 751–755. 10.1038/nphys4140.

[ref10] Liu X.; Hao Z.; Watanabe K.; Taniguchi T.; Halperin B. I.; Kim P. Interlayer fractional quantum Hall effect in a coupled graphene double layer. Nat. Phys. 2019, 15, 893–897. 10.1038/s41567-019-0546-0.

[ref11] Li J. I. A.; Shi Q.; Zeng Y.; Watanabe K.; Taniguchi T.; Hone J.; Dean C. R. Pairing states of composite fermions in double-layer graphene. Nat. Phys. 2019, 15, 898–903. 10.1038/s41567-019-0547-z.

[ref12] Kim Y.; Yun H.; Nam S. G.; Son M.; Lee D. S.; Kim D. C.; Seo S.; Choi H. C.; Lee H. J.; Lee S. W.; Kim J. S. Breakdown of the interlayer coherence in twisted bilayer graphene. Phys. Rev. Lett. 2013, 110, 096602 10.1103/PhysRevLett.110.096602. 23496735

[ref13] Cao Y.; Fatemi V.; Demir A.; Fang S.; Tomarken S. L.; Luo J. Y.; Sanchez-Yamagishi J. D.; Watanabe K.; Taniguchi T.; Kaxiras E.; Ashoori R. C.; Jarillo-Herrero P. Correlated insulator behaviour at half-filling in magic-angle graphene superlattices. Nature 2018, 556, 80–84. 10.1038/nature26154. 29512654

[ref14] Cao Y.; Fatemi V.; Fang S.; Watanabe K.; Taniguchi T.; Kaxiras E.; Jarillo-Herrero P. Unconventional superconductivity in magic-angle graphene superlattices. Nature 2018, 556, 43–50. 10.1038/nature26160. 29512651

[ref15] Yankowitz M.; Chen S.; Polshyn H.; Zhang Y.; Watanabe K.; Taniguchi T.; Graf D.; Young A. F.; Dean C. R. Tuning superconductivity in twisted bilayer graphene. Science 2019, 363, 1059–1064. 10.1126/science.aav1910. 30679385

[ref16] Sharpe A. L.; Fox E. J.; Barnard A. W.; Finney J.; Watanabe K.; Taniguchi T.; Kastner M. A.; Goldhaber-Gordon D. Emergent ferromagnetism near three-quarters filling in twisted bilayer graphene. Science 2019, 365, 605–608. 10.1126/science.aaw3780. 31346139

[ref17] Lu X.; Stepanov P.; Yang W.; Xie M.; Aamir M. A.; Das I.; Urgell C.; Watanabe K.; Taniguchi T.; Zhang G.; Bachtold A.; MacDonald A. H.; Efetov D. K. Superconductors, orbital magnets and correlated states in magic-angle bilayer graphene. Nature 2019, 574, 653–567. 10.1038/s41586-019-1695-0. 31666722

[ref18] Serlin M.; Tschirhart C. L.; Polshyn H.; Zhang Y.; Zhu J.; Watanabe K.; Taniguchi T.; Balents L.; Young A. F. Intrinsic quantized anomalous Hall effect in a moiré heterostructure. Science 2020, 367, 900–903. 10.1126/science.aay5533. 31857492

[ref19] Saito Y.; Ge J.; Watanabe K.; Taniguchi T.; Young A. F. Independent superconductors and correlated insulators in twisted bilayer graphene. Nat. Phys. 2020, 16, 926–930. 10.1038/s41567-020-0928-3.

[ref20] Stark R. W.; Falicov L. M. Chapter VI Magnetic Breakdown in Metals. Prog. Low Temp. Phys. 1967, 5, 235 10.1016/S0079-6417(08)60124-9.

[ref21] Blount E. I. Bloch Electrons in a Magnetic Field. Phys. Rev. 1962, 126, 1636 10.1103/PhysRev.126.1636.

[ref22] Pippard A. B. Magnetic Breakdown. Proc. R. Soc. 1962, A270, 1.

[ref23] Moon P.; Koshino M. Energy spectrum and quantum Hall effect in twisted bilayer graphene. Phys. Rev. B: Condens. Matter Mater. Phys. 2012, 85, 195458 10.1103/PhysRevB.85.195458.

[ref24] Kim Y.; Herlinger P.; Moon P.; Koshino M.; Taniguchi T.; Watanabe K.; Smet J. H. Charge inversion and topological phase transition at a twist angle induced van Hove singularity of bilayer graphene. Nano Lett. 2016, 16, 5053 10.1021/acs.nanolett.6b01906. 27387484

[ref25] Cao Y.; Luo J. Y.; Fatemi V.; Fang S.; Sanchez-Yamagishi J. D.; Watanabe K.; Taniguchi T.; Kaxiras E.; Jarillo-Herrero P. Superlattice-induced insulating states and valley-protected orbits in twisted bilayer graphene. Phys. Rev. Lett. 2016, 117, 116804 10.1103/PhysRevLett.117.116804. 27661712

[ref26] Sanchez-Yamagishi J. D.; Taychatanapat T.; Watanabe K.; Taniguchi T.; Yacoby A.; Jarillo-Herrero P. Quantum Hall effect, screening, and layer-polarized insulating states in twisted bilayer graphene. Phys. Rev. Lett. 2012, 108, 076601 10.1103/PhysRevLett.108.076601. 22401231

[ref27] Sanchez-Yamagishi J. D.; Luo J. Y.; Young A. F.; Hunt B. M.; Watanabe K.; Taniguchi T.; Ashoori R. C.; Jarillo-Herrero P. Helical edge states and fractional quantum Hall effect in a graphene electron–hole bilayer. Nat. Nanotechnol. 2017, 12, 118–122. 10.1038/nnano.2016.214. 27798608

[ref28] Kim Y.; Park J.; Song I.; Ok J. M.; Jo Y.; Watanabe K.; Taniguchi T.; Choi H. C.; Lee D. S.; Jung S.; Kim J. S. Broken-symmetry quantum Hall states in twisted bilayer graphene. Sci. Rep. 2016, 6, 38068 10.1038/srep38068. 27905496 PMC5131475

[ref29] Kim K.; DaSilva A.; Huang S.; Fallahazad B.; Larentis S.; Taniguchi T.; Watanabe K.; LeRoy B. J.; MacDonald A. H.; Tutuc E. Tunable moiré bands and strong correlations in small-twist-angle bilayer graphene. Proc. Natl. Acad. Sci. U. S. A. 2017, 114, 3364–3369. 10.1073/pnas.1620140114. 28292902 PMC5380064

[ref30] Zibrov A. A.; Spanton E. M.; Zhou H.; Kometter C.; Taniguchi T.; Watanabe K.; Young A. F. Even-denominator fractional quantum Hall states at an isospin transition in monolayer graphene. Nat. Phys. 2018, 14, 930–935. 10.1038/s41567-018-0190-0.

