# Supplementary material for: Odd Integer Quantum Hall States with Interlayer Coherence in Twisted Bilayer Graphene
Source: Nano Lett. 2021 May 6;21(10):4249–54. doi: 10.1021/acs.nanolett.1c00360 (PMC8289309; doi:10.1021/acs.nanolett.1c00360)
Supplement: Supplementary file 1 — nl1c00360_si_001.pdf [file nl1c00360_si_001.pdf]

Supporting Information for "Odd integer quantum Hall states with interlayer coherence in twisted bilayer graphene"

Youngwook Kim[1,2], Pilkyung Moon[3], Kenji Watanabe[4], Takashi Taniguchi[5], and Jurgen H. Smet[1*]

[1]*Max-Planck-Institut für Festköperforschung, 70569 Stuttgart, Germany*

[2]*Department of Emerging Materials Science, DGIST, 42988 Daegu, Korea*

[3]*Arts and Sciences, NYU Shanghai, Shanghai 200122, China and NYU-ECNU Institute of Physics at NYU Shanghai, Shanghai 200062, China*

[4]*Research Center for Functional Materials, National Institute for Materials Science, Tsukuba 305-0044, Japan*

[5]*International Center for Materials Nanoarchitectonics, National Institute for Materials Science, Tsukuba 305-0044, Japan*

S1. Sample fabrication and gating details

The van der Waals heterostructure consists of a stack of seven layers fabricated with the dry stamp pick-up transfer method[S1] supported by a thermally oxidized Si substrate: graphitic bottom gate, hBN, twisted bilayer, hBN, graphitic top gate and hBN cap layer. The hBN layers and the graphite layers have a thickness between 5 and 10 nm. The twisted bilayer is identical to the one that has been studied in Ref. S2. However, the device reported at that time suffered from a short circuit between the front- and back-gate. This short-circuit was successfully removed by performing an additional e-beam lithography step and an etching step. The availability of a top and bottom gate allows for the application of a displacement electric field D between the two graphene layers. In the color rendition of the longitudinal resistance recorded across the plane spanned by the total density n_{tot} and the displacement electric field D plotted in Fig. 1c of the main text, n_{tot} follows from $(C_{TG}V_{TG}+C_{BG}V_{BG})/e-n_0$. Here, V_{TG} and V_{BG} are the top and bottom gate voltages, and C_{TG} and C_{BG} are the capacitances per unit area. The elementary charge is denoted as e. The residual charge when both gate voltages are set to zero is referred to as n_0. The above capacitances were determined experimentally from the gate voltage dependencies of the Shubnikov-de Haas oscillation frequencies. The displacement electric field is calculated from the expression $D/\varepsilon_0 = (C_{TG}V_{TG}-C_{BG}V_{BG})/2 - D_0/\varepsilon_0$. Here, ε_0 is the permittivity of free space and D_0 is the residual electric field originating from residual charges in both layers.

S2. Top- and back-gate voltage dependence of the longitudinal resistance

Panel a of Fig. S1 illustrates the dependence of the four terminal longitudinal resistance on the back-gate voltage for fixed zero top-gate voltage, whereas panel b shows data as a function of the top-gate voltage for zero back-gate voltage. These measurements were performed at 1.3 K. As mentioned in the main text, three main features are visible. They occur when the chemical potential aligns with the Dirac point at the $\overline{K}$-symmetry point of the Brillouin zone (middle) and when the mini-band around zero energy is completely occupied (right feature) or emptied (left feature). The latter two cases occur near the $\overline{\Gamma}$-point and also result in a resistance peak as it resembles charge neutral conditions. The density change required to fully occupy the miniband provides an estimate of the twist angle, which equals approximately 2°.

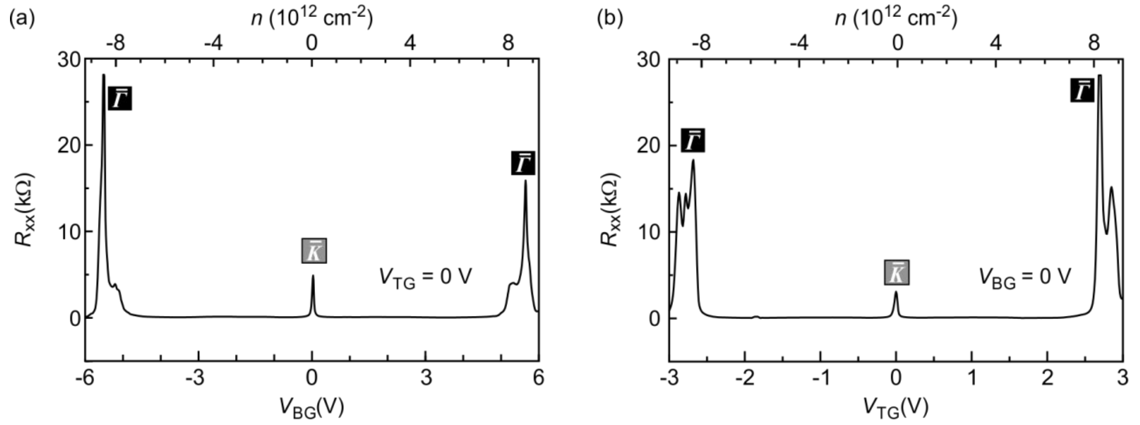

Fig. S1 | Dependence of the longitudinal resistance with top-gate voltage and back-gate voltage in the absence of a magnetic field. (a) The resistance curve for a varying back gate (density) with a zero bias top gate. (b) As in (a), but when sweeping the top gate voltage at a fixed back gate voltage of 0 V instead.

S3. Weakening of the reentrant quantum Hall behavior at $\nu_{tot} = 1$

With increasing magnetic field/density, the resistance features that appear as a result of reentrant quantum Hall behavior at $\nu_{tot} = 1$ when moving away from zero displacement field eventually weaken and vanish as can be seen in Fig. S2. This is similar to what is discussed in the main text for $\nu_{tot} = 3$. The arrows in Fig. S2 mark the resistance peaks separating the incompressible ground state due to Bose-Einstein condensation with interlayer coherence from quantum Hall behavior at non-zero displacement due the condensation of both layers separately in different integer quantum Hall states. The reentrant resistance features remain clearly visible up to 11 T, but weaken significantly at higher fields.

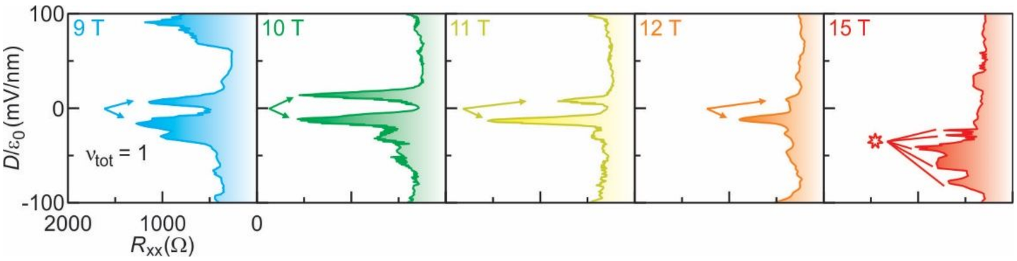

Figure S2 | Longitudinal resistance at $\nu_{tot} = 1$ as a function of D/ε_0 for different values of the magnetic field (from left to right): 9, 10, 11, 12, and 15 T. All windows have the same abscissa covering a longitudinal resistance from 0 to 2 kΩ. The red star with arrows in the panel for 15 T marks resistance features that result from low conductivity of the graphene contact legs as explained in section S3. This artefact is most pronounced when the areas not covered by the bottom gate enter the true insulating state at $\nu_{tot} = 0$.[S3,S4] It causes strong fluctuations of the resistance.

S4. Effect of graphene electrode regime with asymmetry gate tuning

The device that we focused on in this manuscript has two graphitic gates, however these gates do not have the same size. More specifically, the top graphite gate covers a larger area of the twisted graphene bilayer than the bottom graphite gate. This is schematically illustrated in panel a of Fig. S3. As a result, part of the graphene bilayer cannot be controlled by the bottom gate. This is the case here in areas of the bilayer that serve as contact legs to the rectangular Hall bar shape. These areas may turn highly resistive, for instance near the charge neutrality point, when the miniband is completely full or empty or when an incompressible ground state such as the $\nu = 0$ state forms in these areas. In Fig. 1(a) such incidents where contacts become poor are visible as parallel diagonal streaks of high resistance. The effect can also be seen in Fig 3(a) and Fig S3(b).

This issue has been discussed previously in the literature studying the fractional quantum Hall effect in bilayer graphene.[S5] In that report, the top and bottom gate were patterned with electron beam lithography to ensure an identical shape of both the top and bottom gate. The graphene based contact legs inevitably still have some area not covered by the gate. However, it is possible to use the doped Si substrate back gate to modify the filling and make sure these areas are conducting well. While in the device presented here, a Si back gate is also available, it is unfortunately not possible to ensure that the entire contact leg area is conducting well, since in regions where the two graphitic gates do not overlap, different densities/fillings are imposed. Fig. S3(b) is an extended version of the color map shown in Fig 3 of the main text covering both electron and hole densities. Although the hole side shows similar quantum Hall states and reentrant quantum Hall transitions, highly resistivity contact leg areas produce diagonal features near zero displacement field. As a result, the quantum Hall features and their transitions are less clear on the hole side than on the electron side.

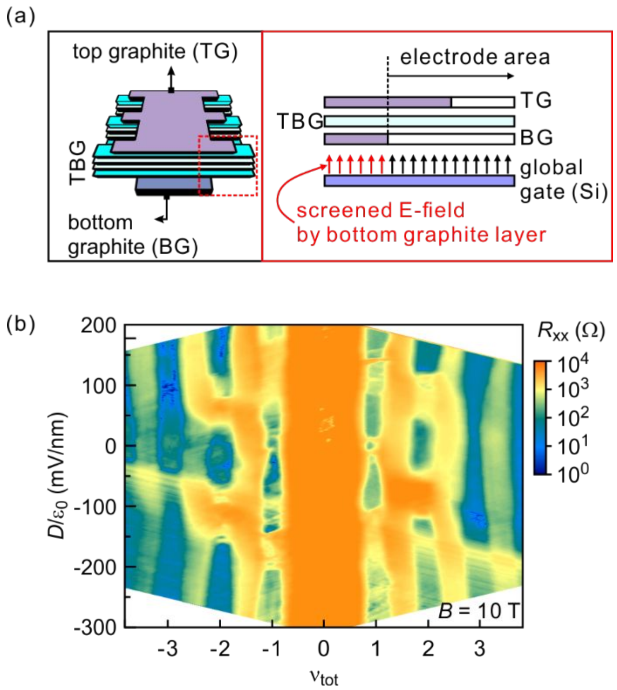

Figure S3 | (a) Left: Schematic of the device. Right: Schematic illustrating the different overlap of the top (TG) and bottom (BG) graphitic gates with the twisted bilayer. The area of the twisted bilayer not covered by the bottom nor the top gate can always be made conducting by applying a suitable voltage to the second, global back gate formed by the doped silicon substrate. Here, typically $|V_{global}| > 50$ V. The active device area itself is not influenced by this global gate voltage, since the electric field is screened by the bottom gate. (b) Color map of the longitudinal resistance as a function of ν_{tot} and displacement field. This figure is the same as Fig 3(a) in the main text, but covers a larger range of total filling factor including hole doping.

S5. Magnetic breakdown in a bilayer with a 2° twist angle

At first sight, it may be surprising that the interlayer coherent states is already suppressed at relatively low density and field (10 T). Usually, magnetic breakdown is prominent for Landau levels close to the van Hove singularity. However, in a bilayer with a 2° twist angle the van Hove singularity already occurs at ±0.025 eV and is much closer to the charge neutrality point (i.e., Dirac point energy, E=0) than the band edges (≈±0.1 eV). Magnetic breakdown starts well before the electron density is such that the chemical potential reaches the van Hove singularity. and a small magnetic breakdown probability may hamper the formation of the BEC state. Figure S4 shows the energy spectrum for the bilayer with a 2° twist angle of 2° plotted against the normalized magnetic field B/B_0 (abscissa) and the energy in eV (ordinate). We refer to Ref. S6 for more details about this calculation. For this system B_0 equals 96.2 T, and hence an externally applied field of 10 T corresponds to a normalized field of about 0.10. The first Landau level already exhibits a finite "band width" below B/B_0=0.10. This can be interpreted as evidence for magnetic breakdown between the electron orbits from the two layers (see also Fig. 6 in Ref. S6). As a result the decoupled nature of the layers is lost quickly and the system can no longer maintain the BEC state.

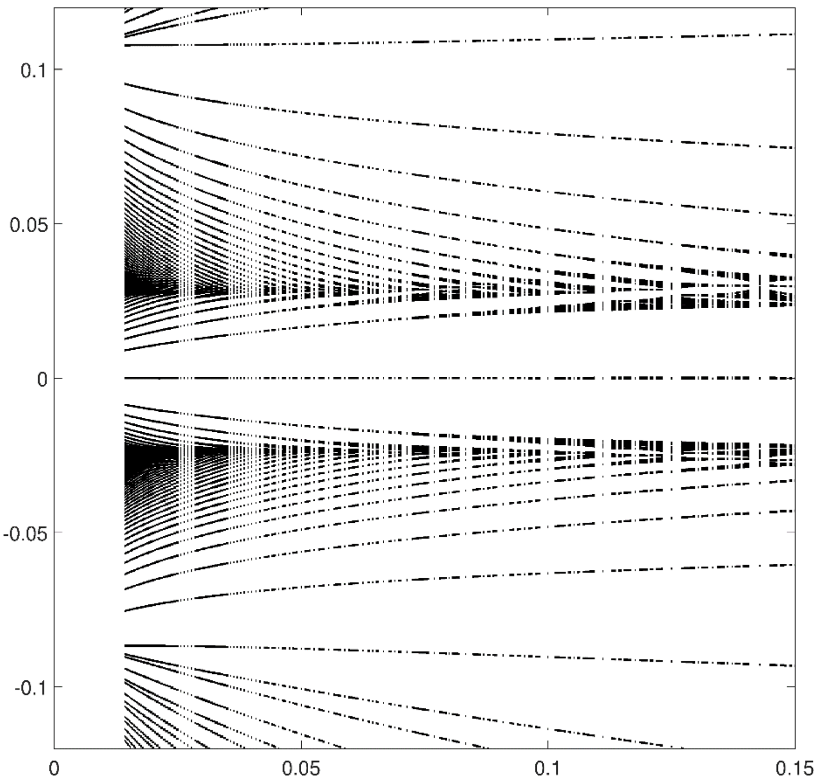

Figure S4| Energy spectrum of twisted bilayer graphene with a twist angle of 2° plotted against the normalized magnetic field B/B_0 (lateral axis, B_0=96.2 T) and energy [eV] (vertical axis).

References for Supporting Information

[S1] L. Wang, I. Meric, P. Y. Huang, Q. Gao, Y. Gao, H. Tran, T. Taniguchi, K. Watanabe, L. M. Campos, D. A. Muller, J. Guo, P. Kim, J. Hone, K. L. Shepard, C. R. Dean, One-dimensional electrical contact to a two-dimensional material. *Science* **342**, 614-617 (2013)

[S2] Y. Kim, P. Herlinger, P. Moon, M. Koshino, T. Taniguchi, K. Watanabe, J. H. Smet, *Nano Lett.* **16**, 5053 (2016).

[S3] Y. Zhang, Z. Jiang, J. P. Small, M. S. Purewal, Y. W. Tan, M. Fazlollahi, J. D. Chudow, J. A. Jaszczak, H. L. Stormer, and P. Kim, *Phys. Rev. Lett.* **96**, 136806 (2006).

[S4] D. S. Lee, V. Skákalová, R. T. Weitz, K. von Klitzing, and J. H. Smet, *Phys. Rev. Lett.* **109**, 056602 (2012).

[S5] P. Maher, C. R. Dean, A. F. Young, T. Taniguchi, K. Watanabe, K. L. Shepard, J. Hone and P. Kim, *Nat. Phys* **9**, 154-158 (2013)

[S6] Pilkyung Moon and Mikito Koshino, *Phys. Rev. B* **85**, 195458 (2012)
